# School modality, race and ethnicity, and mental health of U.S. adolescents during the COVID-19 pandemic

**DOI:** 10.1186/s13034-024-00773-5

**Published:** 2024-07-13

**Authors:** Vijaya Tamla Rai, Linnea Irina Laestadius, Celeste Campos-Castillo

**Affiliations:** 1 Department of Sociology and Anthropology, Montana State University, Bozeman, MT USA; 2 Joseph J. Zilber College of Public Health, University of Wisconsin-Milwaukee, Milwaukee, WI USA; 3 Department of Media and Information, Michigan State University, East Lansing, MI USA

**Keywords:** Virtual learning, Remote learning, Anxiety, Depression, COVID-19

## Abstract

**Background:**

While minoritized ethnoracial groups were most likely to be in online learning during the COVID-19 pandemic, the impact of these ethnoracial disparities on adolescent mental health is unclear. Since past studies do not directly examine whether the association between school modality and self-reported mental health outcomes varied by race and ethnicity among U.S. adolescents during the COVID-19 pandemic, this study addresses the gap.

**Methods:**

Adolescents aged 13 to 17 years old (*n* = 510) were surveyed for self-reports of anxiety and depression symptoms using the 4-item Patient Health Questionnaire during Spring 2021. Seemingly unrelated regressions were used to estimate the differential association between school modality and mental health by respondents’ race and ethnicity.

**Results:**

Estimates without interaction between school modality and race and ethnicity suggested that Latino respondents reported a significantly higher frequency of depressive symptoms than their White counterparts (b = 0.459; *p* < 0.05). Similarly, the estimates without the interaction suggested respondents reporting hybrid learning had a higher frequency of depressive symptoms than in-person learning (b = 0.504; *p* < 0.05). Estimates with interaction between school modality and race and ethnicity suggested fully online learning was associated with poorer mental health only among White respondents and better mental health among Black respondents. Among adolescents attending school fully online, Black adolescents reported fewer mental health symptoms than their White counterparts (anxiety, b =– 1.364; *p* < 0.05, and depression, b =– 1.647; *p* < 0.05).

**Conclusions:**

Fully online learning may have benefitted the mental health of Black adolescents during the COVID-19 pandemic, perhaps because it buffered racial discrimination and social anxiety in schools. Additional interventions should be explored to promote in-person school environments that better support the mental health of Black adolescents. Moreover, prioritizing equitable access to broadband internet will provide better access to online learning and ensure positive mental health, particularly for adolescents from minoritized ethnoracial groups during instances of future pandemics. Future research should continue to consider the race and ethnicity of adolescents to promote mental well-being in schools across learning modalities.

## Background

The COVID-19 pandemic significantly altered the schooling of about 55 million K-12 students in the United States [1]. The shift to online learning raised concerns about mental distress, particularly for adolescents from minoritized ethnoracial groups [2–4]. Despite the growing number of studies evaluating this claim [3, 5, 6], to our knowledge, no study has directly examined whether school mode differentially affected the mental health of adolescents from minoritized ethnoracial groups [7]. Therefore, we ask: Does the association between school modality and mental health vary by race and ethnicity of adolescents?

This is important to examine since adolescents from minoritized ethnoracial groups tended to be more likely in online learning than their White counterparts, particularly among high school-aged populations [1, 8]. For instance, a national survey of 567 adolescents administered during the Fall of 2020 in the United States found that 68% of Black respondents and 69% of Hispanic respondents were in the online mode of learning compared to only 48% of White respondents in online learning [1]. At the beginning of the pandemic, this was because they were more likely to attend schools in areas hit hard by COVID-19 cases [4]. As the pandemic continued and decisions about school openings became politicized, they were still more likely to be in online learning than their White counterparts [6]. For instance, a nationally representative survey of 2,152 U.S. adolescents during Spring 2021 found that online learning was more common than in-person learning for Black respondents (17% versus 11%) and Hispanic respondents (33% versus 20%), whereas White respondents (39% versus 63%) were less likely to be in online learning than in-person learning [8].

Whether the differential tendency to be in online learning among adolescents of different ethnoracial groups contributed to mental health inequalities is poorly understood. Studies on adolescent mental health and mode of school instruction suggested that online learning students reported poorer mental health than in-person learning students [1, 6]. While virtual learning was challenging for all students, ethnoracially minoritized students disproportionately experienced challenges due to barriers such as lack of access to highspeed internet and computer devices [8]. While many studies included race and ethnicity as a control variable in the regression models estimating the influence of school modality on mental health, the interaction between school modality and race and ethnicity was not examined [7]. For instance, one study found the association between school modality and mental health outcomes varied significantly by child age and family income level [6]. The researchers adjusted for race and ethnicity but did not estimate an interaction between school modality and race and ethnicity. Thus, using data collected in Spring 2021 from U.S. high school adolescents, we tested whether the association between school modality and reports of anxiety and depression varied by race and ethnicity.

## Methods

### Participants

The National Opinion Research Center (NORC) at the University of Chicago administered the cross-sectional online survey from March to May 2021 to 540 adolescents (784 invited, 68.9% completion rate) aged 13 to 17 years from their AmeriSpeak Teen Panel. NORC’s AmeriSpeak Teen Panel is a nationally representative survey infrastructure designed to collect data from U.S. teens. Black and Latino respondents were oversampled to improve the comparison of patterns by different ethnoracial groups. We present an analysis of the 510 respondents who stated they attended high school during the survey. 30 respondents not attending high school during the survey were dropped. NORC administered consent and assent procedures online, with parents/guardians providing consent and adolescents providing assent.

### Measures

Using the 4-item Patient Health Questionnaire (PHQ-4), respondents reported the frequency of experiencing mental distress (0 = not at all to 3 = nearly every day) during the two weeks preceding the survey [9]. The PHQ-4 is validated for use among adolescents as a screener for anxiety and depression and has shown measurement invariance across ethnoracial groups [9–11]. Two items represented anxiety symptoms, and the other two represented depressive symptoms. Items were summed to create two indices, each ranging between 0 and 6, with larger numbers representing higher levels of mental distress.

We used four categories for self-reported race and ethnicity: White, Black, Latino, and Other. The Other category included those who identified as Asian, American Indian, and multi-racial, which we combined into one category because of the small numbers. We used four categories for self-reported school modality: in-person, fully online, hybrid, and home-schooled. We included as covariates respondents’ sex, age, metropolitan area, access to broadband internet, annual household income, household size, geographic region, and number of hours spent per day direct messaging [2, 6].

### Statistical analysis

Statistical analyses were conducted with Stata 17.1. In Stata, sureg command for seemingly unrelated regressions (SUR) was applied. SUR allows simultaneous estimations of anxiety and depression with error terms correlated. This is an advantage over estimating anxiety and depression with two independent ordinary least squares regression because it accounts for measured (e.g., race and ethnicity, sex) and unmeasured (e.g., academic performance) factors shaping both [12]. We estimated SUR twice, first without the key interactions between school modality and race and ethnicity, to replicate how prior studies estimate the mental health of adolescents during the pandemic. In doing so, we showed the comparability of our sample with previous studies. In the second SUR estimates, we advanced this prior work by adding the interaction terms. All estimates were adjusted for the covariates. Significance tests were two-tailed and set at 0.05.

## Results

### Characteristics of respondents

The sample (*n* = 510, 55% female) included 53% White, 15% Black, 22% Latino, and 10% Other respondents. About 31% of the respondents reported being in-person (*n* = 156), 37% fully online (*n* = 188), 25% hybrid (*n* = 130), and 7% home-schooled (*n* = 36).

### Regression without interaction

The first two columns in Table 1 show the results of seemingly unrelated regressions when estimating mental health without interaction terms. The models show the association between school modality and reports of mental health when adjusting for the race and ethnicity of the respondents. Respondents reporting hybrid learning had higher depressive symptomatology than those reporting in-person learning (b = 0.504; *p* < 0.05). We found no significant difference in mental health outcomes between U.S. adolescents reporting in-person learning and other learning modalities (i.e., fully online and home-schooled).

**Table 1:** Results of seemingly unrelated regressions without and with interactions between school modality and race/ethnicity

	Model 1		Model 2		Model 3		Model 4	
	Anxiety		Depression		Anxiety		Depression	
	b (s.e.)	p	b (s.e.)	p	b (s.e.)	p	b (s.e.)	p
Race								
*(Reference: White)*								
Black	– 0.127 (0.275)	0.645	– 0.164 (0.247)	0.507	0.435 (0.562)	0.439	0.728 (0.503)	0.147
Latino	0.112 (0.237)	0.638	0.459* (0.213)	0.031	0.596 (0.428)	0.164	0.967* (0.383)	0.012
Other	– 0.157 (0.291)	0.588	– 0.04 (0.261)	0.877	0.253 (0.651)	0.698	– 0.166 (0.582)	0.776
School modality								
*(Reference: In-person learning)*								
Fully online	– 0.338 (0.22)	0.124	0.079 (0.198)	0.690	0.121 (0.287)	0.674	0.554* (0.256)	0.031
Hybrid	0.333 (0.238)	0.161	0.504* (0.213)	0.018	0.328 (0.303)	0.279	0.401 (0.27)	0.138
Home-schooled	0.005 (0.359)	0.989	0.295 (0.322)	0.359	0.297 (0.474)	0.530	0.809 (0.423)	0.056
Fully online interaction								
Black					– 1.364* (0.667)	0.041	– 1.647** (0.596)	0.006
Latino					– 0.998 (0.54)	0.065	– 0.982* (0.483)	0.042
Other					– 0.412 (0.809)	0.610	– 0.199 (0.723)	0.783
Hybrid interaction								
Black					0.185 (0.759)	0.807	– 0.249 (0.679)	0.714
Latino					– 0.182 (0.603)	0.763	– 0.127 (0.539)	0.814
Other					– 0.451 (0.795)	0.571	0.627 (0.711)	0.378
Home-schooled interaction								
Black					– 0.326 (0.944)	0.730	– 1.186 (0.844)	0.160
Latino					– 1.107 (0.972)	0.255	– 1.61 (0.869)	0.064
Other					– 2.369 (1.547)	0.126	– 1.737 (1.383)	0.209
Female sex	0.909*** (0.172)	0.000	0.569*** (0.154)	0.000	0.923*** (0.172)	0.000	0.564*** (0.154)	0.000
Age > 15 y vs. < 15 y	0.203 (0.173)	0.241	– 0.412 (0.155)	0.790	0.192 (0.173)	0.268	– 0.068 (0.155)	0.661
Metro area vs. Rural area	– 0.157 (0.238)	0.509	– 0.043 (0.214)	0.841	– 0.179 (0.237)	0.449	– 0.081 (0.212)	0.702
Access to broadband internet	0.166 (0.289)	0.566	– 0.073 (0.259)	0.777	0.184 (0.287)	0.520	– 0.032 (0.256)	0.902
Annual household income								
*(Reference: < $30,000)*								
$30,000 to under $60,000	0.365 (0.253)	0.150	0.308 (0.227)	0.176	0.359 (0.251)	0.152	0.325 (0.224)	0.147
$60,000 to $100,000	0.358 (0.265)	0.176	0.456 (0.237)	0.055	0.312 (0.265)	0.239	0.424 (0.236)	0.073
$100,000 or more	0.093 (0.271)	0.732	0.021 (0.243)	0.931	0.4 (0.272)	0.883	0.016 (0.243)	0.948
Household size	0.035 (0.756)	0.644	-0.003 (0.068)	0.971	0.067 (0.076)	0.380	0.029 (0.068)	0.670
Geographic region								
*(Reference: Midwest)*								
Mid-Atlantic	– 0.182 (0.554)	0.742	– 0.231 (0.497)	0.642	– 0.276 (0.55)	0.616	– 0.326 (0.492)	0.508
East North Central	– 0.426 (0.521)	0.413	– 0.212 (0.467)	0.649	– 0.428 (0.517)	0.408	– 0.239 (0.462)	0.605
West North Central	0.112 (0.561)	0.842	0.089 (0.503)	0.860	0.021 (0.557)	0.969	– 0.056 (0.498)	0.910
South Atlantic	– 0.322 (0.519)	0.535	– 0.331 (0.466)	0.477	– 0.397 (0.516)	0.441	– 0.417 (0.462)	0.366
East South Central	– 0.441 (0.584)	0.449	– 0.625 (0.524)	0.232	– 0.609 (0.581)	0.295	– 0.798 (0.519)	0.125
West South Central	– 0.588 (0.557)	0.291	– 0.339 (0.499)	0.497	– 0.743 (0.554)	0.180	– 0.499 (0.495)	0.313
Mountain	– 0.509 (0.555)	0.358	– 0.426 (0.498)	0.392	– 0.582 (0.549)	0.290	– 0.522 (0.491)	0.288
Pacific	– 0.191 (0.534)	0.721	– 0.217 (0.479)	0.650	– 0.202 (0.531)	0.704	– 0.279 (0.475)	0.555
Daily hours spent in direct messaging	0.289*** (0.065)	0.000	0.243*** (0.058)	0.000	0.287*** (0.065)	0.000	0.246*** (0.058)	0.000
Intercept	2.405** (0.737)	0.001	2.571*** (0.661)	0.000	2.204** (0.738)	0.003	2.361*** (0.659)	0.000
R-squared	0.132		0.115		0.153		0.143	

The sample size was 510

*p <.05; **p <.01; ***p <.001

As for differences by race and ethnicity, only one appeared. Latino respondents reported a higher frequency of depressive symptoms than their White counterparts (b = 0.459; *p* < 0.05). There was no significant difference between Black and White respondents’ reports of mental health.

### Regression with interaction

**Fig. 1:**
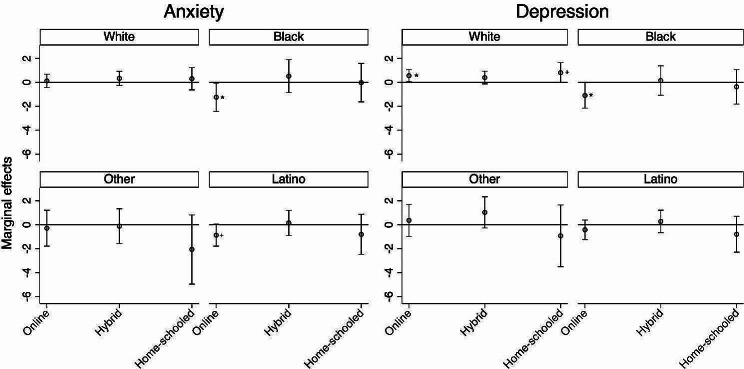
Marginal effects of school modality (reference: in-person learning) on anxiety and depression with 95% Confidence Interval. *p < 0.05; +p < 0.10

 As shown in the last two columns of Table 1, there was a significant interaction between school modality and race and ethnicity for anxiety and depression. Figure 1 shows the marginal effects of school modality on anxiety and depression with 95% Confidence Interval among White, Black, Latino, and Other adolescents. Values that do not cross zero indicate a significant difference in reports of mental distress compared to those in in-person learning. Compared to adolescents in in-person learning, Black respondents in fully online learning reported lower anxiety and depressive symptomatology, while White respondents in fully online learning reported higher depressive symptomatology.

Estimates without the interaction terms showed that adolescents in hybrid learning reported higher depressive symptomology than those in in-person learning. But, as shown in the bottom sections of the table, there was no significant interaction between hybrid learning and race/ethnicity. Likewise, there was no significant interaction between home-schooled learning and race/ethnicity for mental health.

## Discussion

When the COVID-19 pandemic hit the United States in March 2020, restrictions on daily lives, including school closures, raised concerns about the mental well-being of children and adolescents [5, 7, 13]. Starting in the fall of 2020, schools began resuming in-person learning, but adolescents from ethnoracially minoritized groups tended to remain in online learning, which raised alarms about ethnoracial mental health disparities [1, 2, 4, 14]. Past studies consistently reported that there were larger proportions of adolescents from minoritized ethnoracial groups attending schools fully online compared to their White counterparts. However, whether this differential tendency of ethnoracially minoritized adolescents to be in online learning was associated with disproportionate mental health harms for ethnoracially minoritized adolescents has been contested [3, 5, 6, 8].

On the one hand, some studies found that attending school online during the pandemic was associated with poor mental health and suggested that there were disproportionate mental health consequences for adolescents from minoritized ethnoracial groups [1, 6]. These studies primarily related school closures and online modes of learning to psychological distress among adolescents due to increased feelings of loneliness, confusion, affective challenges, and fear of SARS-CoV-2 infection. Since ethnoracially minoritized adolescents were more likely to experience school closures and online learning, those studies assumed that Black adolescents experienced more mental health difficulties [1, 6]. On the other hand, some studies suggested that despite disproportionately attending schools online during the pandemic, ethnoracially minoritized adolescents reported either comparable or better mental health than their White counterparts [3, 5]. For instance, one study found that during the pandemic, the prevalence of having seriously considered attempting suicide was lower among Black students than their White counterparts [5]. But, to our knowledge, no study directly examined how the association between school modality and mental health outcomes varied by race and ethnicity among U.S. adolescents during the COVID-19 pandemic [7]. Therefore, our study addresses this gap.

We found online learning was negatively associated with White adolescents’ mental health but positively associated with Black adolescents’ mental health. The positive association between online learning and Black adolescents’ mental health may reflect how online learning relieved Black adolescents from stress due to racist encounters (e.g., disciplinary infractions), social anxiety, concerns about adequate safety measures for resuming in-person learning, and elevated risks of contracting SARS-CoV-2 [3, 4, 13, 15, 16].

In the United States, race and ethnicity correlate with trauma exposure during the pandemic. For example, past studies found that compared to White adolescents, Black adolescents were at increased risk of losing multiple loved ones and subsequent posttraumatic anxiety-related disorders [16]. During the pandemic, Black families and children experienced significantly higher rates of SARS-CoV-2 infections, hospitalizations, and deaths [4]. In addition, due to institutional and structural racism, the majority of Black adolescents in public schools historically attended schools with concentrated poverty. Those historically underresourced schools were likely to have poor school building quality, such as outdated HVAC (heating, ventilation, and air conditioning) systems, raising concerns for SARS-CoV-2 transmissions [15]. Therefore, the online modality perhaps reduced or buffered Black adolescents from adverse mental health outcomes influenced by their concerns about inadequate safety measures in in-person learning, SARS-CoV-2 transmission from schools to families at home, and racism-related anxiety [4, 14–17].

Consistent with previous studies of U.S. adolescents [2, 17], adolescents reporting hybrid learning reported higher levels of depressive symptomatology than those reporting in-person learning. The association did not seem to vary by race and ethnicity. The finding may reflect that hybrid learning often varies on the number of in-person and online learning days, which may increase stress from managing daily routines [2].

As we continue to live with the impacts of COVID-19, concerns will remain about the long-term effects of school modality on the mental health of adolescents. This study identified the moderating influence of race and ethnicity on the association between pandemic school modality and adolescents’ mental health, but additional research is warranted. It should also be stressed that ensuring reliable access to broadband internet and computer devices among students from minoritized ethnoracial groups must be a priority for future pandemic response efforts to avoid any learning decline or negative mental health consequences associated with lacking resources for online learning [7, 8].

Future research on how returning to in-person schooling after the pandemic impacted adolescents’ mental health and how that varied by race and ethnicity is needed to improve our understanding of the relationship between school modality and mental health among adolescents from different ethnoracial groups. These efforts can help inform ethnoracially equitable intervention strategies to support the mental well-being of children and adolescents across different learning modalities in the future.

### Limitations

This study has several limitations. First, responses from this English-language self-reported survey may not represent the broader U.S. adolescent population, specifically because it likely lacks perspectives from the 5 million English Learners in U.S. K-12 school systems who were disproportionately impacted by digital access gaps during the COVID-19 pandemic [18]. Second, the study lacks details on respondents’ mental health before the pandemic. Had data been available before the pandemic, we could have formally examined whether online learning buffered mental health consequences due to racist encounters in in-person learning for Black adolescents. Lastly, reliance on a cross-sectional design impedes inferring causality between school modality and mental health by race and ethnicity.

Despite these limitations, this study has implications for adolescents, families, school administrators, mental health practitioners, and policymakers. It is critically important to mitigate racial and ethnic disparities in adolescents’ mental health. Black adolescents reporting online learning reported less anxiety and depression during the pandemic. As with benefits accruing from expanding telehealth modalities [13], findings demonstrated that adopting technologies to facilitate virtual access to schools during the pandemic appears to have equity-promoting effects. Given disparities in access to broadband internet during this time, it is important to ensure resources are in place to enable any potential equity-promoting effects [8, 19]. Policymakers and mental health practitioners need to recognize the differential association between school modality and mental health by race and ethnicity rather than blanketing the positive association between in-person schooling and adolescents’ mental health and the negative association between online learning and adolescents’ mental health.

As most schools have returned to in-person learning, interventions should be explored to promote in-person school environments that better support the mental health of Black adolescents [1, 17]. Further research is needed to concretely identify the specific pathways through which online learning supported mental health and determine if benefits can be replicated across modalities. Lastly, results from this study can assist families and schools in mitigating ethnoracial disparities in adolescent mental health associated with school modality during future pandemics and even now, since online learning remains in lesson plans, at least in part.

## Conclusion

This study adds a snapshot of how the relationship between school modality and mental health outcomes among U.S. adolescents during the pandemic varied by race and ethnicity. The findings highlight that the online learning school modality was only negatively associated with White adolescents’ mental health, while it was positively associated with Black adolescents’ mental health. Our study suggests that as schools have resumed in-person learning, families, and schools should work to create school environments that better promote mental health among minoritized racial groups. Lastly, prioritizing reliable access to broadband internet and computer devices will improve online learning accessibility and promote mental well-being, particularly for adolescents from minoritized ethnoracial groups during instances of future pandemic.

## Data Availability

The data that support the findings of this study are available from the third author, upon reasonable request.
